# Comprehensive review on Fanconi anemia: insights into DNA interstrand cross-links, repair pathways, and associated tumors

**DOI:** 10.1186/s13023-025-03896-w

**Published:** 2025-07-30

**Authors:** Chenyan Fang, Zhoujun Zhu, Jun Cao, Jun Huang, Yipeng Xu

**Affiliations:** 1 Department of Gynecologic Oncology, Zhejiang Cancer Hospital, Hangzhou Institute of Medicine (HIM), Chinese Academy of Sciences, Hangzhou, 310022 Zhejiang China; 2 Department of Gynecology, Campus Virchow Klinikum, Charité-Universitätsmedizin Berlin, 10117 Berlin, Germany; 3 Department of Obstetrics and Gynecology, Ruijin Hospital, Shanghai Jiao Tong University School of Medicine, Shanghai, 200025 China; 4 Department of Head and Neck and Rare Oncology, Zhejiang Cancer Hospital, Hangzhou Institute of Medicine (HIM), Chinese Academy of Sciences, Hangzhou, 310022 Zhejiang China; 5 Zhejiang Key Laboratory of Geriatrics and Geriatrics Institute of Zhejiang Province, Affiliated Zhejiang Hospital, Zhejiang University School of Medicine, Hangzhou, 310058 China; 6 Department of Urology, Zhejiang Cancer Hospital, Hangzhou Institute of Medicine (HIM), Chinese Academy of Sciences, Hangzhou, 310022 Zhejiang China

## Abstract

Fanconi anemia (FA) is a rare genetic disorder caused by defects in the repair of DNA interstrand crosslinks (ICLs)—highly toxic lesions that impede essential processes like DNA replication and transcription, leading to severe genome instability. Clinically, FA presents with a broad spectrum of symptoms, including progressive bone marrow failure, congenital abnormalities, and an elevated predisposition to various malignancies, particularly acute myeloid leukemia and squamous cell carcinomas. This review provides a comprehensive overview of both the endogenous and exogenous sources of ICLs and the DNA repair pathways responsible for their resolution, with a primary focus on the FA pathway. We also discuss the tumorigenic consequences of FA pathway deficiencies, highlighting the molecular mechanisms that contribute to the heightened cancer risk observed in FA patients.

## Introduction

Fanconi anemia (FA) is a rare autosomal or X-linked recessive genetic disorder characterized by early bone marrow failure with pancytopenia, which may progress to aplastic anemia. Other common features include endocrine and developmental issues (e.g., growth hormone deficiency, delayed puberty, diabetes), congenital abnormalities (e.g., thumb/radial defects, skin pigmentation, kidney and genital anomalies, microcephaly), reproductive dysfunction, and cancers susceptibility (e.g., myelodysplastic syndrome (MDS), acute myeloid leukemia (AML), head and neck or genital squamous cell carcinoma (SCC), breast/ovarian cancer). Some patients also have neurodevelopmental delays, learning difficulties, and psychological stress, requiring comprehensive care and psychological support [1, 2]. FA affects approximately 1 in 136,000 newborns, with males being slightly more susceptible than females [3] and occurring in all ethnic groups.

**Fig. 1:**
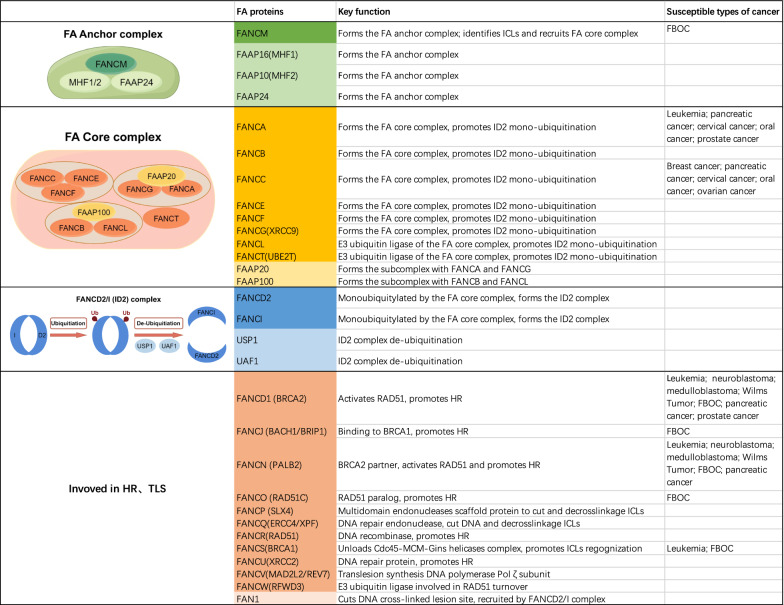
Fanconi anemia proteins and the key function: Twenty-two FA proteins and several important FA pathway-related proteins, as well as their complexes that play key roles in the FA pathway. *FA, Fanconi anemia; HR, homologous recombination; TLS, translesion synthesis; ICLs, interstrand crosslinks; FBOC, familial breast and ovarian cancer*

Currently, 22 FA genes have been identified **(**Fig. 1), including FANCA, B, C, D1 (BRCA2), D2, E, F, G (XRCC9), I, J (BACH1/BRIP1), L, M, N (PALB2), O (RAD51C), P (SLX4), Q (ERCC4/XPF), R (RAD51), S (BRCA1), T (UBE2T), U (XRCC2), V (MAD2L2/REV7), W (RFWD3), as well as FA-associated proteins, including FAAP10 (MHF2), FAAP16 (MHF1), FAAP20, FAAP24, FAAP100, UHRF1/2, USP1/UAF1, FAN1, etc. [4, 5]. Notably, some studies have suggested that FANCM may not be a bona fide FA gene, as classical FA phenotypes are not observed in individuals with biallelic FANCM mutations [6, 7]. FA diagnosis primarily relies on low-cost next-generation sequencing (NGS), functional tests such as hypersensitivity assays to ICL agents (e.g., diepoxybutane, mitomycin C) and chromosome breakage analysis also remain crucial [8–11]. Data from various countries indicate that FANCA mutations are the most common in FA patients, accounting for 60–70%, followed by FANCC (7–15%) and FANCG (10%). One study reported 27% of the patients carried co-mutations in FANC genes, highlighting the important role of the co-occurrence of different genetic mutations in amplifying FA phenotypes [12]. Additionally, 3–8.6% of patients may lack mutations in known FA genes, suggesting the existence of yet unidentified genes [13–18].

FA genes encode proteins involved in DNA damage repair pathways, particularly in the repair of interstrand crosslinks (ICLs). ICLs are covalent linkages between bases on opposite DNA strands, which hinder DNA replication and transcription. Mutations in FA genes lead to defects in the FA pathway, resulting in impaired repair of ICLs, which in turn causes chromosome breakage and rearrangements, ultimately leading to chromosomal instability [13], which is particularly detrimental to cells that replicate and divide rapidly, such as hematopoietic stem cells. The defects can potentially affect all systems in the body. Compared with the general population, FA patients are more likely to develop cancer at an early age, with the median age of SCC onset is 33 years [19]. Moreover, the risk of head and neck, esophagus, and anogenital SCCs is hundreds to thousands of times higher in FA patients [20].

Hematopoietic stem cell transplantation (HSCT) serves as an effective treatment for FA, significantly improving the survival rates of FA patients. Therefore, fertility defects and the increased incidence of tumors have become critical research directions for FA patients [21, 22]. Recent advances in gene therapy offer promising alternatives, including the autologous transplantation of gene-corrected hematopoietic stem cells via lentiviral vectors, as demonstrated in a European phase I/II trial (NCT03157804), where 62.5% (5 out of 8) of patients showed clinical benefits without preconditioning or genotoxicity [23].

This review primarily summarizes the research findings on the FA DNA damage repair pathway and the mechanisms of FA-related tumors, aiming to provide references and inspiration for subsequent researchers.

### DNA-ICLs

In organisms, DNA is covalently combined with compounds and their metabolites to produce DNA adducts, which exist in multiple forms, including base adducts, cyclic adducts, DNA intrastrand crosslinks, DNA ICLs, and DNA protein crosslinks. ICLs can vary in their proportion of the total DNA lesions induced by crosslinking inducers, depending on the specific crosslinking agent, dosage, and experimental conditions used. For example, in the case of cisplatin, intrastrand crosslinks are much more prevalent than ICLs, with ICLs accounting for less than 5% of its induced DNA adducts. Similarly, Psoralen is about three times more likely to form monoadducts with pyrimidine bases than ICLs [24, 25]**.** The FA DNA repair pathway primarily mediates the repair of DNA-ICLs, thus a comprehensive understanding of the origin of ICLs and DNA alterations is crucial for studying FA-related mechanisms [26, 27]. Additionally, it has been reported that FA repair pathway may also be involved in the repair of DNA protein crosslinks, but the specific mechanism is unclear and needs further study [28, 29].

**Fig. 2:**
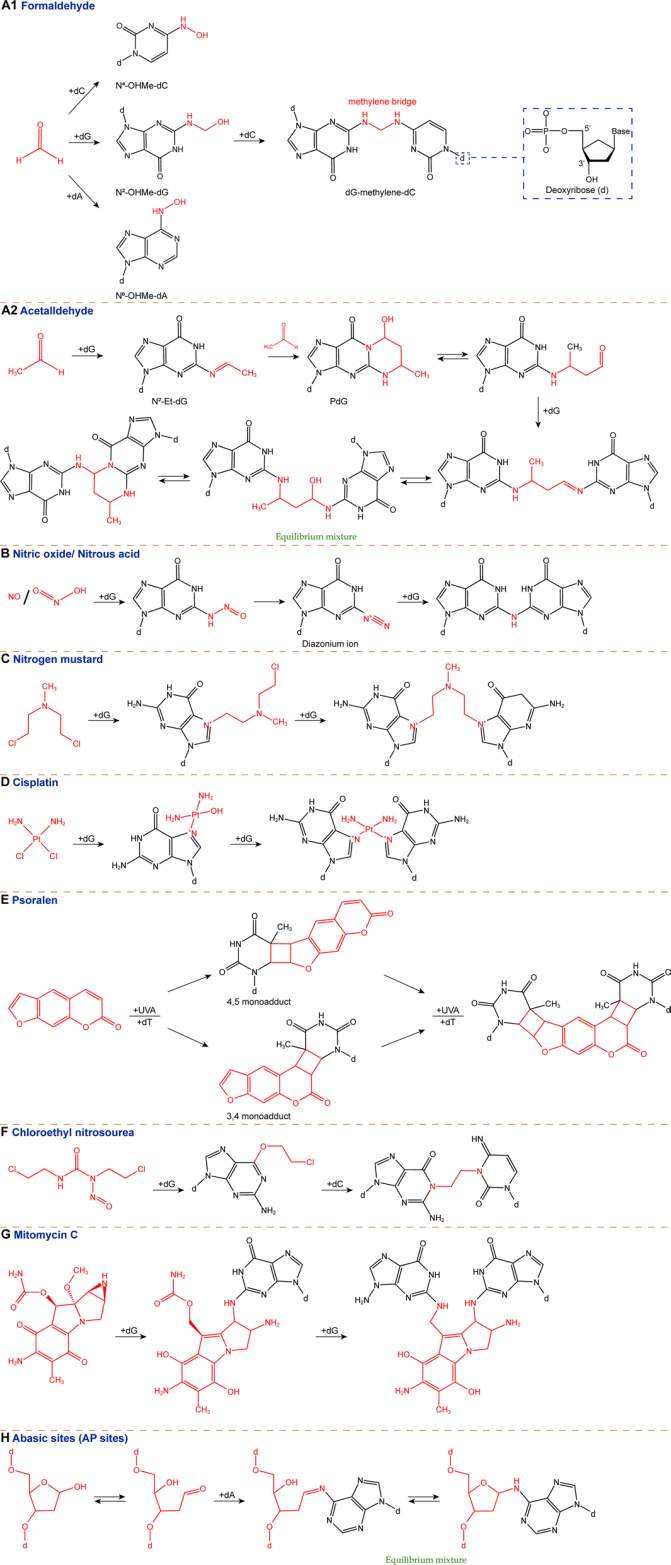
The origin and different types of ICLs: **A1** Formaldehyde. **A2** Acetaldehyde. **B** Nitric oxide/Nitrous acid. **C** Nitrogen mustard. **D** Cisplatin. **E** Psoralen. **F** Chloroethyl nitrosourea. **G** MMC (Mitomycin C). **H** Abasic sites (AP sites). *dG, deoxyguanine; dC, deoxycytosine; dA, deoxyadenine; AP, apurinic/apyrimidini*

DNA cross-linking occurs when various endogenous or exogenous crosslinkers react with two nucleotides of DNA to form a covalent link between them, resulting in the formation of ICLs as illustrated in Fig. 2.

### Endogenous crosslinking inducers

Endogenous cross-linking inducers refer to cellular metabolites or endogenous compounds in organisms, such as lipid peroxidation products, amino acid metabolites, demethylation products induced by cytochrome P450 enzyme systems, free radicals, etc. [30]. The primary components are aldehydes, including formaldehyde, acetaldehyde, malondialdehyde, acrolein, crotonaldehyde, and 4-hydroxynonenal, as well as nitrogen compounds generated by nitrate/nitrite metabolism, such as nitric oxide (NO) and nitrous acid [31, 32].

Formaldehyde reacts with the amino groups on guanine, adenine, and cytosine, forming base adducts: N^2^-hydroxymethyl-deoxyguanine (N^2^-OHMe-dG), N^6^-hydroxymethyl-deoxyadenine (N^6^-OHMe-dA), and N^4^-hydroxymethyl-deoxycytosine (N^4^-OHMe-dC). Formaldehyde can also covalently link guanine, adenine, and cytosine to opposing DNA strands by forming methylene bridges, thereby forming ICLs of any combination of these three bases, such as: dG-methylene-dG, dA-methylene-dA, and dG-methylene-dC, etc. (Fig. 2A1). Acetaldehyde reacts with guanine to form the unstable base adducts: N^2^-ethylidene-deoxyguanine (N^2^-Et-dG), which then forms cyclic adducts with another molecule of acetaldehyde, known as N^1^, N^2^-propano-deoxyguanine (PdG). These cyclic adducts can further lead to ICLs of guanines in the DNA double helix (equilibrium mixtures of cyclic and extended forms) (Fig. 2A2). Human hematopoietic stem and progenitor cells are protected from endogenous aldehyde damage by high expression of aldehyde dehydrogenase-2 (ALDH2), which detoxifies formaldehyde and acetaldehyde, and alcohol dehydrogenase 5 (ADH5), which detoxifies formaldehyde. If aldehydes have already induced DNA ICLs in cells, they are primarily repaired through the FA pathway [32, 33]. Nitrogen compounds such as nitric oxide and nitrite also form cross-links between guanines in DNA **(**Fig. 2B**)** [34, 35].

### Exogenous crosslinking inducers

Exogenous crosslinking inducers mainly include aldehydes, nitrogen mustards, platinum-based chemotherapeutic agents, psoralens, chloroethyl nitrosoureas and compounds produced by bacteria, such as mitomycin C (MMC), azinomycin B, and colibactin [36].

Nitrogen mustards (e.g., chlorambucil, cyclophosphamide, ifosfamide, mechlorethamine, melphalan), which are used in the treatment of lymphoma, multiple myeloma, melanoma, ovarian cancer, chronic lymphocytic leukemia, can produce aziridinium ions. These ions further alkylate N^7^ and O^6^ sites of dG, N^3^ and N^1^ sites of dA, and N^3^ site of dC, resulting in cross-links between dG–dG, dG–dA, and dA–dA through interactions with the opposite DNA strands **(**Fig. 2C**)** [31, 37, 38].

Platinum-based chemotherapeutic agents (e.g., cisplatin, carboplatin, oxaliplatin), which are used to treat ovarian cancer, cervical cancer, breast cancer, non-small cell lung cancer, and other cancers, undergo ligand exchange in cells where their two chloride ligands are replaced by water molecules. These activated complexes form adducts with the N^7^ site of dG. Then most commonly resulting in intrastrand cross-links with a second dG or dA on the same DNA strand, or ICLs with dG on the opposite strand that cause more severe DNA distortion. (Fig. 2D**)** [39].

Psoralens are composed of pyrone and aromatic fused furan, which are derived from plants and are used as photosensitizers in the treatment of skin diseases. In combination with ultraviolet irradiation, they can be used to treat vitiligo, psoriasis, cutaneous T-cell lymphoma. Psoralens first insert non-covalently between the base pairs of the DNA double helix. Upon activation by ultraviolet light, the pyrone and furan rings of the same psoralen molecule bind to the 5,6 double bonds of two deoxythymidines on opposite DNA strands, respectively, forming cyclobutane adducts. This results in slight distortion of the DNA (Fig. 2E**)** [39–41].

Similarly, the anticancer drugs chloroethyl nitrosoureas (e.g., carmustine and lomustine) initially produce adducts at the O^6^ site of dG, which then cyclize with the N^1^ site and subsequently connect to the N^3^ site of dC on the opposite DNA strands via ethylene linkages, forming dG–dC ICLs (Fig. 2F**)** [42].

MMC, derived from Streptomyces caespitosus, is mainly used to treat bladder cancer and esophageal cancer. After enzymatic reduction, it reacts with the N^2^ site of dG to form an adduct, which then forms a mildly distorting dG–dG ICL with a dG on the opposite strand** (**Fig. 2G**)** [43, 44]. Similarly derived from Streptomyces, Azinomycin B reacts with the N^7^ site of dG to cross-link DNA [45]. Colibactin, the metabolite of Escherichia coli strains that is thought to be associated with colorectal cancer, reacts with the N^3^ site of dA to produce DNA cross-links [46, 47].

In addition, ICLs can also be generated by DNA damage, such as abasic-apurinic/apyrimidinic (AP) sites caused by anti-tumor treatments (e.g., radiotherapy and chemotherapy). These AP sites induce ICLs between dA residues of the DNA duplex, existing as an equilibrium mixture between the open- and closed- ring forms of deoxyribose (Fig. 2H**)** [48].

### Repair of ICLs

The repair of DNA ICLs primarily relies on the FA/BRCA pathway, Additionally, endonuclease VIII-like 3 (NEIL3) and acetaldehyde pathways can repair ICLs independently via nucleotide excision and translesion synthesis (TLS) mechanisms [49].

The degree of DNA strand deformation and distortion caused by ICLs, along with the structure of ICLs, the timing of their generation (cell-cycle phase), and their location in the genome, influences the choice of repair pathway [36].

### FA/BRCA pathway

Due to various endogenous and exogenous factors such as ICLs, replication stress occurs, leading to the slowing or even stalling of DNA replication fork progression [50, 51]. Persistent replication stress results in replication fork collapse, leading to DNA double-strand break (DSB). Some researchers have found that the FA repair pathway essentially represents a unique homologous recombination (HR) pathway, the break-induced replication (BIR) pathway. FA proteins facilitate the cleavage and restart of stalled replication forks to resume replication progression [52]. There are two replication-dependent repair models of ICLs through the FA pathway: forks convergence model and single forks traverse model.

#### Forks convergence model (Fig. 3A)

**Fig. 3:**
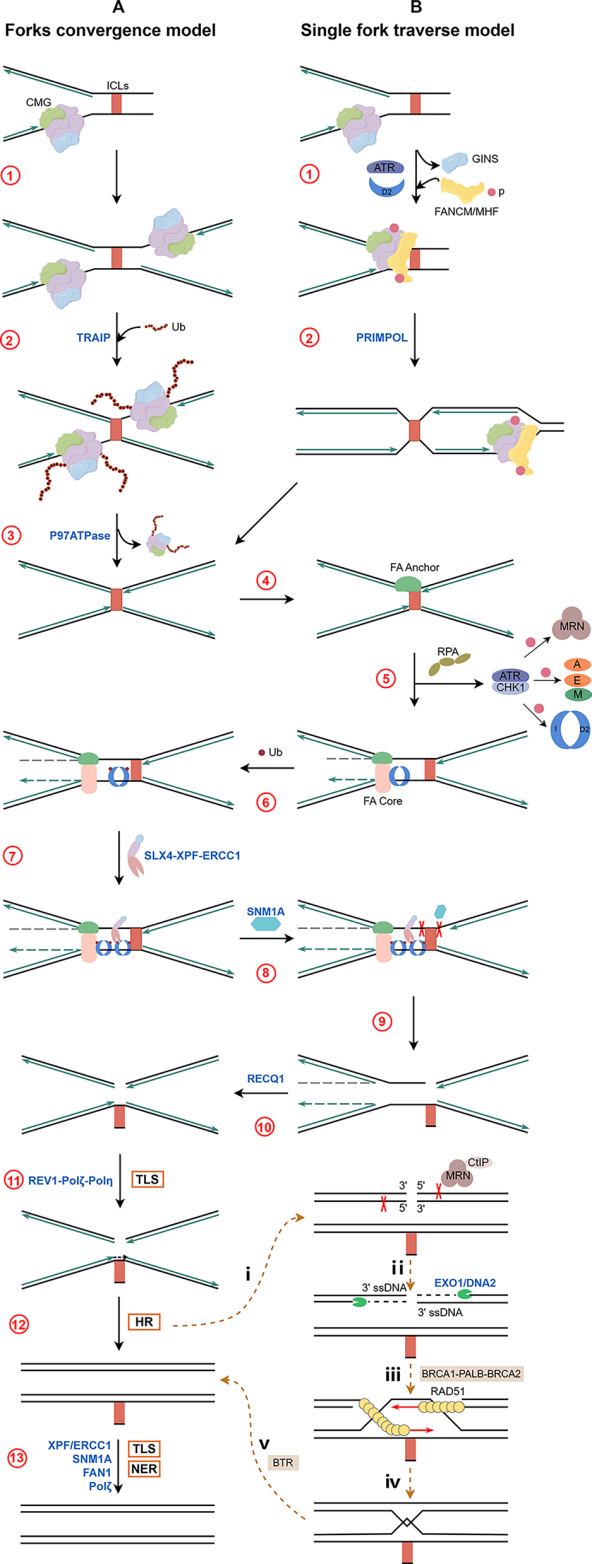
FA/BRCA pathway: A Forks convergence model, ① Two DNA fokes convergence; ② TRAIP (E3 ubiquitin ligase)-mediated ubiquitination of the CMG helicase; ③ P97 ATPase—mediated removal of ubiquitinated CMG helicase from the ICLs site, then forms the X-shaped structures; ④ Anchor complex (FANCM-FAAP24-MHF1/2) detects the ICLs and binds to replication fork; ⑤ RPA recruitment on the DNA lesions, activates ATR-CHK1 (leading to fork slowing, fork reversal and the phosphorylation of FANCM/A/E/I/D2, MRN complex), which promotes the assembly of FA core complex and ID2 complex on chromatin; ⑥ Ubiquitination of ID2 complex and release from the FA core complex onto the DNA adjacent to the ICLs (the de-ubiquitination of ID2 complex mediated by USP1-UAF1 complex is essential for repair); ⑦ Ubiquitinated ID2 complex recruits scaffold proteins for various DNA endonucleases (FANCP/SLX4-XPF/FANCQ-ERCC1 complex), recruitment and ubiquitination of new ID2 complexs; ⑧ Recruitment of SNM1A, which cooperates with XPF perform the 3'flap incision and 5'flap incision on both sides of the ICLs site; ⑨ ICLs unhooking, leads to DNA substrates suitable for HR and TLS; ⑩ Fork reversal restoration prior to TLS (the enzymes involved are unclear); ⑪ REV1-Pol ζ (REV3) -Pol η complex-mediated TLS on the leading strand, which produces the intact DNA duplex to provide a template for HR of the lagging strand; ⑫ DSBs repaired by HR, ***i*** MRN-CtIP complex-mediated DSB 5’ end resection, results in 3’ ssDNA; ***ii*** EXO1/DNA2-mediates extending of 3’ ssDNA; ***iii*** FANCS/BRCA1- FANCN/PALB2- FANCD1/BRCA2 complex-mediated recruitment of FANCR/RAD51 to ssDNA, produce the RAD51 nuclear fibril, promotes the strand invasion; ***iv*** Strand invasion forms a DHJ; ***v*** BTR complex-mediated DHJ resolution; ⑬ Removement of the monoadducts through NER and then DNA gap filling through TLS. B Single fork traverse model, ① Replication fork encounters ICLs triggers replisome remodeling, promotes ATR activation, then the phosphorylated FANCM/MHF and MRN complex leads to the release of GINS from the CMG complex, opens the CMG ring; ② CM complex bypasses the ICLs and continues unwinding DNA, following PRIMPOL-mediated DNA replication outside of ICLs, finally forms the X-shaped structures. *FA, Fanconi anemia; CMG, CDC45-MCM2-7-Gins; ICLs, interstrand crosslinks; RPA, replication protein A; ATR, ataxia telangiectasia mutated and Rad3-related; CHK1, checkpoint kinase 1; TLS, translesion synthesis; HR homologous recombination; DSB double-strand break; MRN, MRE11-RAD50-NBS1; EXO1, exonuclease 1; DNA2, DNA synthesis defective 2; DHJ, double Holliday junction; BTR, BLM-TOPO3α-RMI1-RMI2*

##### Formation of X-shaped structures

In early studies of ICLs repair, the main model was the single fork model, where a single replication fork completed ICLs unhooking, TLS, HR, and excision of the monoadduct. However, the single fork repair model is difficult to distinguish ICLs from transient obstacles caused by stable nucleoprotein complexes and other structures, which can easily lead to unnecessary replication stress.

In 2008, it was proposed that two DNA replication forks are involved in the repair of ICLs. Studies on site-specific ICLs repair in *Xenopus* egg extracts revealed that convergence of two replication forks triggers the ubiquitination of the mini-chromosome maintenance 2–7 (MCM2–7) complex in the CMG (CDC45-MCM2–7-GINS) helicase mediated by the E3 ubiquitin ligase TRAF-interacting protein (TRAIP) (Fig. 3A**①-②**) [53, 54]. Subsequently, the ubiquitinated CMG helicase is removed from the ICLs site through the action of the p97 ATPase, forming X-shaped structures (Fig. 3A**③**) [55]. Then, one of the two replication forks undergoes reversal, while the other fork adjacent to the ICL undergoes incisions [56]. However, due to the lack of identified enzymes involved in fork reversal, it is still unclear whether replication fork reversal is a necessary step in ICL repair.

##### ICLs recognition and ID2 complex ﻿ubiquitination

Firstly, the anchor complex composed of FANCM, FAAP24, and MHF1/2 binds to a stalled replication fork (Fig. 3A**④**). FANCM-dependent translocation promotes replication fork remodeling, leading to the recruitment of replication protein A (RPA) to DNA damage sites [57]. The single-stranded DNA (ssDNA) wrapped by RPA further activates the ataxia-telangiectasia and Rad3-related protein (ATR)-checkpoint kinase 1 (CHK1) dependent checkpoint response, promoting fork slowing, RAD51-mediated fork reversal, and the phosphorylation of multiple FA-related proteins, including FANCM/A/I/D2, MRN (MRE11-RAD50-NBS1) complex phosphorylated by ATR, and FANCE phosphorylated by CHK1 [58–60]. Phosphorylated FANCM promotes the assembly of the core complex on chromatin. Subsequently, through the interaction between the RING domain of FANCL and the interface of the ID2 complex, as well as the binding of FANCE to FANCD2, the ID2 complex is recruited to the core complex (Fig. 3A**⑤**). Additionally, some studies suggest that after the formation of ICLs, ubiquitin-like with PHD and ring finger domains 1 (UHRF1) can be rapidly recruited to ICLs sites through its SET and RING finger associated (SRA) domain, which promotes the recruitment of FANCD2 to ICLs [61]. The binding of ID2 complex to DNA promotes the closure of the heterodimer, exposing the K561 mono-ubiquitination site of FANCD2 and facilitating its ubiquitination by FANCL. Subsequently, ubiquitinated FANCD2 exposes the K523 site on FANCI, leading to its ubiquitination. The mono-ubiquitinated ID2 complex is detached from the FA core complex and locked onto the DNA regions adjacent to ICLs, thereby promoting lesion excision and recruitment of new ID2 complexes [62, 63] (Fig. 3A**⑥**). However, studies also show that the isoleucine 44 (Ile44) motif of ubiquitin, which is essential for interaction with the ubiquitin-binding zinc finger (UBZ) domain of the incision complex, is typically buried. This motif can be exposed by various factors, including conformational changes in the ubiquitin structure or the activity of ubiquitin-modifying enzymes. Further research is still needed to clarify how the Ile44 motif becomes exposed to enable the successful recruitment of the incision complex with UBZ domain [64–66].

However, during the cell's G2/M phase or mitosis, the hyperphosphorylation of FANCM mediated by Polo-like kinase 1 (PLK1) dissociates the FA core complex from the chromosome [67]. Moreover, in the absence of DNA damage such as ICLs, FANCD2 882-898 phospho-clusters mediated by Casein kinase 2 (CK2) inhibits the binding of the ID2 complex to DNA, thereby suppressing the activity of the FA pathway [68]. Ubiquitin specific peptidase 1 (USP1)—ubiquitin associated factor 1 (UAF1) complex is a deubiquitinating enzyme that targets both the ID2 complex and proliferating cell nuclear antigen (PCNA) [69]. Deubiquitination of the ID2 complex is essential for completing ICL repair, as ubiquitination keeps the ID2 complex bound to the DNA strand. Failure to remove ubiquitin and release the DNA leads to sustained activity of the incision complex, which in turn hinders the completion of subsequent repair processes [64]. This is consistent with previous results that knockout of USP1 in chicken DT40 cells and mouse cells result in the persistence of ubiquitylated FANCD2 on chromatin, thereby impairing DNA cross-link damage repair [70, 71]. Conversely, phosphorylation of FANCI serine residues 559 and 565 mediated by ATR protects ubiquitinated ID2 complexe from deubiquitination by the USP1-UAF1 complex, ensuring the proper functioning of the FA pathway [72].

##### ICLs unhooking

Ubiquitinated ID2 complexes recruit FANCP/SLX4 via its UBZ domain to ICLs, then recruit endonucleases to cleave the crosslink damage on the ssDNA, leaving fragments containing ICLs attached to the opposite strand, thereby generating DNA substrates suitable for HR and TLS. Known associated endonucleases include XPF/FANCQ-ERCC1, MUS81–EME1, SLX1, FAN1, and SNM1A. However, recent studies have shown that MUS81–EME1, SLX1, and FAN1 are not essential enzymes for ICL unhooking in the FA pathway, while SLX4-XPF/FANCQ-ERCC1 and SNM1A play important roles in the unhooking process [73–79]. Bric-a-brac, Tramtrack, Broad complex (BTB) and MUS312/MEI9 interaction-like region (MLR) domain of SLX recruit XPF/FANCQ-ERCC1 to perform both the 3’ and 5’ flap incision on either side of the ICLs site. SNM1A acts synergistically with XPF in 5’ flap incision during ICLs repair (Fig. 3A⑦-⑨) [74]. It is worth mentioning that FAN1 possesses 5’−3’ exonuclease activity like SNM1A and a UBZ domain like SLX4 [80]. However, UBZ is not necessary for the initial rapid recruitment of FAN1 to ICLs. FAN1 can repair ICLs independently of the FA pathway [81–84].

##### Translesion synthesis (TLS)

The TLS polymerase complex (REV1-Pol ζ/REV3-Pol η) is recruited to the site of unhooked ICLs on the DNA leading strand, bypasses the lesion, and extends the leading strand to connect it to the Okazaki fragment downstream of the replication fork, thereby creating the intact DNA duplex, providing a template for HR (Fig. 3A⑪) [85]. And the scaffolding function of REV1 can interact with Pol κ, Pol ζ, Pol ι, and Pol η [86]. Studies have reported that the recruitment of TLS complex is mediated by high-fidelity replicative polymerases such as Pol δ and Pol ε, which activate the RAD6–RAD18 ubiquitin ligase complex, leading to the ubiquitination of PCNA with TLS polymerases binding domain [87]. It has also been shown that FANCD2 can bind to Polη, then promote the recruitment of TLS complex [88]. However, more studies tend to indicate the involvement of the FA core complex in recruiting TLS polymerases. For example, in Xenopus egg extracts, it is FANCA binding with REV1 and REV7, not the ID2 complex [89]. FAAP20 with UBZ4 can bind to ubiquitinated REV1, promoting the interaction between the FA core complex and the PCNA-REV1 complex [90]. Before TLS, restoration of fork reversal during ICLs unhooking is required (Fig. 3A⑩). Currently, the enzymes or proteins involved in restoration process are still unclear. It has been reported that human RecQ Like Helicase 1 (RECQ1) can repair replication fork reversal induced by Topoisomerase I (TOP1) inhibitors, and further research is needed to investigate whether RECQ1 helicase plays a similar role in the repair process of ICLs [91].

##### Homologous recombination (HR)

Unhooking of ICLs from the lagging strand template during DNA replication results in DSBs, and the repair of DSBs associated with the FA pathway relies on HR. HR mainly involves three steps: DSB end resection (5’ end DNA degradation), strand invasion, and Holliday junction resolution. Initially, the MRN complex recognizes DSBs, binds to DNA ends, and promotes DNA single strand resection with its cofactor CtBP-interacting protein (CtIP), leading to 5’ end DNA degradation and generating 3’ ssDNA (Fig. 3A⑫i) [92, 93]. Studies have also shown that FANCS/BRCA1, FANCM, and monoubiquitinated FANCD2 can promote the recruitment of CtIP to ICLs [94–96]. Moreover, the FANCJ/BACH1/BRIP1 helicase not only promotes the recruitment of CtIP to DSB sites but also regulates the MRE11 nuclease within the MRN complex to ensure proper end resection [97, 98]. Additionally, Exonuclease 1 (EXO1) or DNA synthesis defective 2 (DNA2) cooperates with Bloom (BLM)/Werner syndrome protein (WRN) to extend 5’ end DNA degradation and elongate 3’ ssDNA overhangs(Fig. 3A⑫ii) [99, 100]. 3’ ssDNA is then coated by RPA, protecting it from nucleases. The coiled-coil domain of BRCA1 recognizes the coiled-coil domain of FANCN/PALB2, which in turn binds to FANCD1/BRCA2 via its WD40 domain, forming the BRCA1-PALB2-BRCA2 complex. This functionally connected complex mediates the recruitment of FANCR/RAD51 to the RPA-coated ssDNA and promotes the formation of RAD51 nucleoprotein filaments [101]. FANCJ interacts with BRCA1 to prevent premature or disorganized recombination during HR through its helicase activity [97, 102]. RAD51 nuclear filament further mediates strand invasion, capturing sequence information from the lagging strand template, pairing with homologous DNA sequences to form a D-loop structure, which extends or connects with another end to complete the repair process (Fig. 3A⑫iii). Strand invasion produces a double Holliday junction (DHJ) (Fig. 3A⑫iv) [103], which is mainly dissolved and decatenated by the BLM-TOPO3α-RMI1-RMI2 (BTR) complex (Fig. 3A⑫v) [36]. In addition, recent studies have demonstrated that two replicative helicase-related MCM complex family members-the MCM8/MCM9 complex is involved in the formation of D-Loop structure mediated by RAD51 protein [104]. And two structural maintenance of chromosomes (SMC) proteins-SMC5/SMC6 complex plays an important role in maintaining the correct pairing of damaged DNA with sister chromatid and ensuring chromosome integrity and stability [105].

If FA pathway is defective, the non-homologous end joining (NHEJ) pathway—another DSB repair mechanism in mammalian cells—is correspondingly upregulated. Simpler than HR, NHEJ directly links DSBs ends by DNA ligase, independent of homologous DNA sequences. Although NHEJ is a more straightforward mechanism, it sometimes results in gene rearrangements, whereas HR, which analyzes homologous sequences of sister chromatid and gathers information lost at the break site, is thought to be error-free.

##### Nucleotide excision repair (NER)

Finally, to complete the repair, the remnants (monoadducts) of the ICLs attached to the DNA strand must be removed by NER, which plays a major role in replication independent ICLs repair [106]. After the DNA duplex is opened and RPA is unloaded from the ssDNA, the remnants of the ICLs are recognized by the NER protein XPC in mammalian cells, then ﻿XPF-ERCC1, SNM1A and FAN1 perform incisions at the lesion site. DNA ﻿gap filling is ﻿completed by TLS mediated by Pol ζ (Fig. 3A⑬). However, the remnants verification, incision and the TLS enzymes involved in the process need further investigation [36, 107].

#### Single fork traverse model

About 20% of the ICLs are located near the replication fork. After encountering ICLs, some replication forks can traverse the lesions and continue DNA replication beyond them. Upon ATR activation and the action of FANCD2, the phosphorylated FANCM/MHF complex interacts with the phosphorylated MCM2-7 complex within the CMG complex. This interaction causes the dissociation of GINS from the CMG complex, opening the CMG ring (Fig. 3B①), thereby bypassing the ICLs and continuing to unwind DNA outside the ICLs. Subsequently, DNA replication resumes outside the ICLs under the mediation of PRIMPOL (Fig. 3B②) [108, 109]. However, direct evidence that CMG complex unwinds DNA beyond ICLs is lacking. Moreover, it has been proposed that ATR activation slow down replication fork progression and simultaneously promotes fork reversal, which may facilitate lesion traverse [58]. Further research is needed to elucidate the mechanism of traverse.

After CMG bypass, subsequent steps—including ICL unhooking, TLS, HR, and NER—are consistent with the convergence pathway. There is a competition between single fork traverse and forks convergence. When adjacent ICLs are located between two prereplication complexes (pre-RCs), or when an ICL is positioned between a telomere and the last pre-RC, forks convergence does not occur. In contrast, forks convergence may be prioritized in cells with very short interorigin distances, as observed in the early frog embryos [36].

### NEIL3 pathway

NEIL3, along with NEIL1 and NEIL2, is a member of the NEIL bifunctional DNA glycosylase family. They cleave the glycosidic bond between deoxyribose and oxidized bases in the first step of base excision repair (BER). In addition to base excision, NEIL1 and NEIL3 can also repair psoralen-ICLs in triple- and quadruple-stranded DNA [24, 110]. In 2016, Semlow et al. investigated the repair mechanism of psoralen-ICLs and AP-ICLs in Xenopus egg extracts, BER enzyme NEIL3 was found to play a crucial role in this process [111]. NEIL3 is rapidly recruited to psoralen-ICLs and AP-ICLs in a poly-ADP-ribose polymerase (PARP)-dependent manner, initiating a repair pathway that is faster than the FA/BRCA pathway and does not generate DSBs. Therefore, under normal conditions, the majority of psoralen-ICLs (about 80%) and AP sites-ICLs are repaired via the NEIL3 pathway, with the FA/BRCA pathway only being utilized in the absence of NEIL3 or in cases where NEIL3-mediated repair fails. Recent studies in human cells have also identified the NEIL3 pathway as the primary mechanism for repairing psoralen-ICLs [40].

**Fig. 4:**
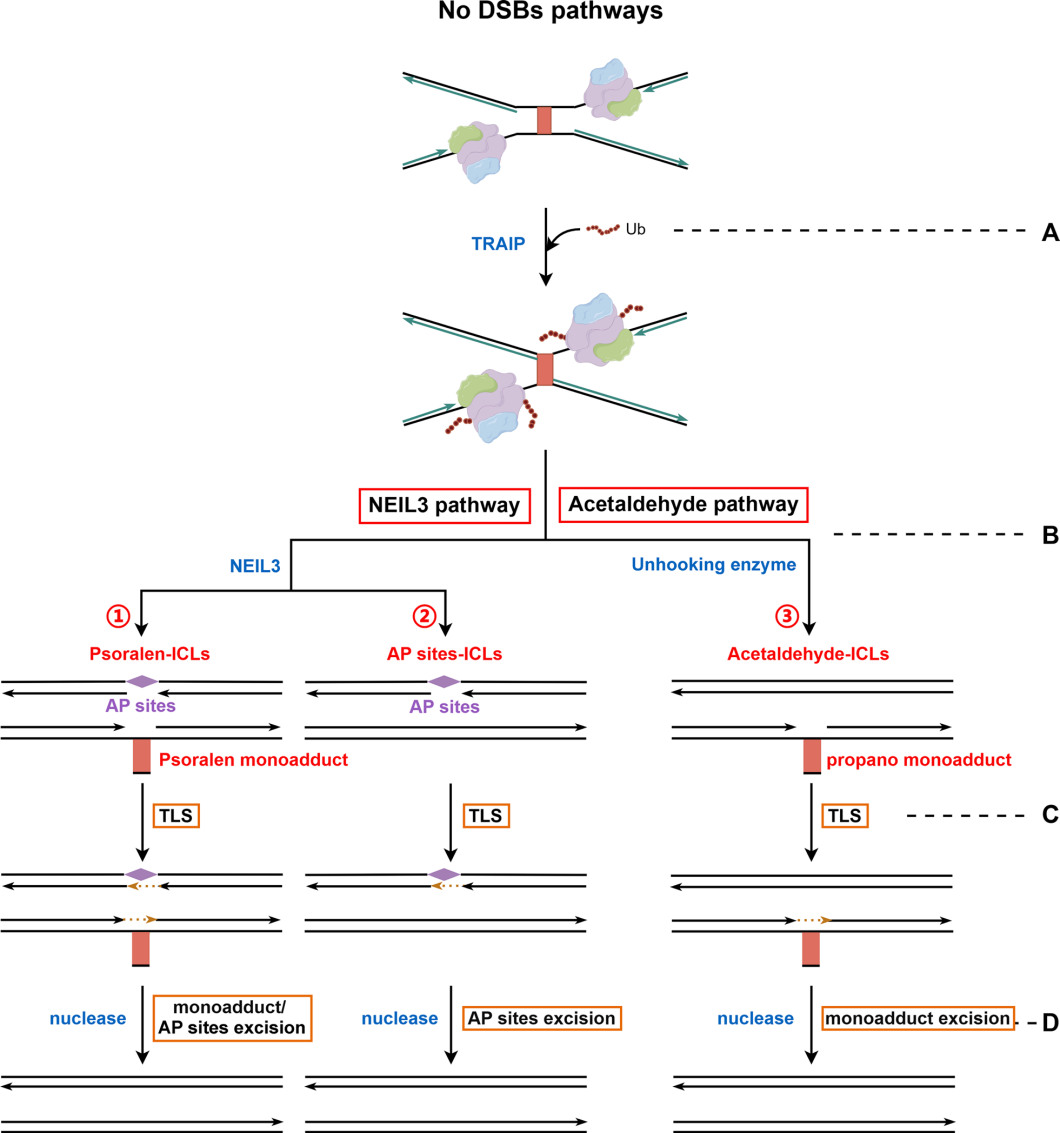
NEIL3 pathway and Acetaldehyde pathway: **A** After two replication forks converging, TRAIP-mediated MCM2-7 complex short ubiquitination in CMG helicase. During this step, if NEIL3 pathway fails, the ubiquitination of CMG complex will be extended, then ICLs are repaired by FA pathway. **B ①** For psoralen-ICLs, NEIL3-mediated incision in N-glycosyl bond, generating one stand with AP sites and opposite strand with psoralen monoadduct; **②** For AP sites-ICLs, NEIL3-mediated reversion of ICLs, generating one stand with AP sites and normal opposite strand; **③** For acetaldehyde-ICLs, Unclear enzyme-mediated the broken within the crosslink itself (reversion of ICLs), generating one normal stand and opposite strand with propano monoadduct. **C** TLS polymerases complex—mediated insertion of nucleotides across from the AP sites, psoralen monoadducts and propano monoadducts. **D** Corresponding nucleases-mediated removal of AP sites, psoralen monoadducts and propano monoadducts. *CMG, CDC45-MCM2-7-Gins; ICLs, interstrand crosslinks; FA, Fanconi anemia; AP, apurinic/apyrimidinic; TLS, translesion synthesis*

NEIL3 pathway, like the FA/BRCA pathway, also relies on TRAIP-mediated ubiquitination of CMG (Fig. 4A), but the difference is that NEIL3 pathway does not require CMG unloading [112]. When the ubiquitin chain on CMG is short, NEIL3 can be recruited via its ubiquitin-binding domain (NPL4-type zinc finger) (Fig. 4B). Different from the FA/BRCA pathway, which cleaves phosphodiester backbone to unhook ICLs, NEIL3 produces incisions in one of the two N-glycosidic bonds that form the crosslink, generating an AP site in one strand and a psoralen monoadduct in the other (Fig. 4B①). However, repair of AP sites-ICLs resulted in normal adenosine in one strand and AP sites in the other strand by reversing the ICLs (Fig. 4B②). Subsequently, cross-lesion repair is mediated by TLS polymerases complex (REV1- Pol ζ) (Fig. 4C), followed by excision repair of AP sites and psoralen monoadducts by corresponding nucleases, but research on these nucleases is still lacking (Fig. 4D).

Additionally, if NEIL3 fails to excise ICLs, as observed in platinum or MMC-ICLs, the ubiquitin chain on CMG continues to elongate. This further recruits p97 ATPase, unloading the CMG helicase from the ICLs site, thereby initiating the FA/BRCA pathway [36].

### Acetaldehyde pathway

Acetaldehyde-ICLs are produced when acetaldehyde produced after alcohol ingestion is not efficiently cleared. Most acetaldehyde-ICLs are repaired through the FA pathway. However, a second, faster, and DSBs-independent repair mechanism has recently been observed in Xenopus egg extract. This mechanism also requires replication fork convergence but operates in an excision-independent manner [113]. Unlike nucleolytic incisions observed during the repair of cisplatin-ICLs or the glycosidic bond cleavage mediated by NEIL3 during the repair of psoralen-ICLs, acetaldehyde pathway involves the breakage within the crosslink itself. Thus, this repair pathway can prevent large-scale genomic instability caused by DNA strand damage or AP sites.

After replication fork convergence and TRAIP-mediated ubiquitination of CMG complex (Fig. 4A), the putative acetaldehyde-ICLs unhooking enzyme induces reversal of the crosslinks (Fig. 4B③), creating an undamaged dG on one strand, and a dG with a propano monoadduct (propano-2'-deoxyguanosine, PdG) on the opposite strand. However, the enzyme for this step remains unclear. Subsequently, consistent with the NEIL3 pathway, there will be cross-lesion repair mediated by TLS polymerases complex (REV1-Pol ζ) (Fig. 4C), and the removal of the propano monoadduct promoted via the corresponding nucleases (Fig. 4D).

## FA repair pathway and cancer

The FA pathway-related proteins play an important role in repairing DNA damage and maintaining genome stability. Mutations or epigenetic changes in FA-related genes may lead to genomic instability and chromosomal abnormalities, thereby increasing the risk of cancer. The cancer incidence rate among FA patients of all age groups is approximately 30%, including hematological malignancies (e.g., MDS, leukemia, etc.) and solid tumors (e.g., SCCs, breast cancer, ovarian cancer, etc.), of which solid tumors account for 30–40% [114, 115]. By the age of 50, the cumulative cancer incidence rate increases to 86% [115].

Additionally, FA pathway plays a critical role in mediating resistance to DNA damage caused by chemotherapy and radiotherapy in tumor cells [116–118]. Non-malignant FA cells are more prone to toxicities from these treatments, FA patients who develop malignancies may face a therapeutic dilemma. Therefore, studying the role of the FA pathway in tumor development and treatment is crucial for understanding the mechanisms of tumorigenesis, predicting reatment responses, and developing novel therapeutic strategies.

### FA repair pathway and hematological malignancies

**Fig. 5:**
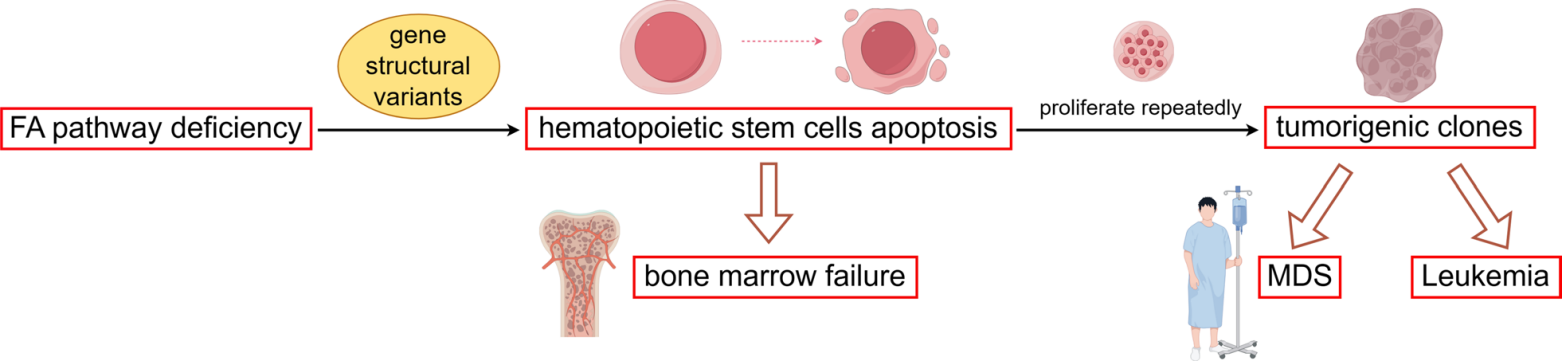
Mechanism of FA pathway deficiency leading to hematologic malignancies: Deficiency of FA repair pathway causes gene structural variants, promotes the apoptosis of hematopoietic stem cells, remaining hematopoietic stem cells repeat proliferation, which results in tumorigenic clones, finally leading to MDS, Leukemia, etc. *FA, Fanconi anemia; MDS, myelodysplastic syndrome*

Defects in the FA repair pathway cause chromosome breakage and gene structural variation, which promote the apoptosis of hematopoietic stem cells and lead to bone marrow failure. According to data from the International Fanconi Anemia Registry (IFAR), the United States National Cancer Institute (NCI), and other major national registries (e.g., India, Spain and Italy), the incidence of bone marrow failure is estimated at 82–96% by age 40, with a median onset age of 6.6–7.4 years [115, 119–122]. The remaining hematopoietic stem cells proliferated repeatedly, resulting in tumorigenic clones. Such clones exhibit higher resistance to apoptosis-inducing cytokines, ultimately resulting in hematologic malignancies (Fig. 5) [123]. Among them, MDS and AML are more common, large registry studies have reported that the incidence of hematologic malignancies in FA patients is 15%−25%, with the average age of onset being 13.3 years and 10.8 years [114, 115, 122, 124, 125].

### FA repair pathway and solid tumors

Due to the effectiveness of hematopoietic stem cell transplantation (HSCT) in treating hematological diseases in FA patients, solid tumors have become the main threat to these individuals. There is still controversy about whether HSCT increases the risk of solid tumors in FA patients. Some studies suggest that the conditioning regimen before HSCT, which includes cyclophosphamide and radiation therapy that can induce DNA cross-linking, may increase the risk of solid tumors [84]. However, some research indicates that there is no significant association between HSCT and the development of solid tumors [114, 115, 126].

FA-related solid tumors can occur in the head and neck, esophagus, breast, ovary, liver, stomach, pancreas, cervix, vulva, anus, bladder, prostate, and other sites [127, 128] The incidence rate is about 13–20%, with the risk increasing significantly with age [115, 122, 125]. Previous studies have shown that germline monoallelic mutations in FANCM, FANCD1/BRCA2, FANCJ/BACH1/BRIP1, FANCN/PALB2, FANCO/RAD51C, and FANCS/BRCA1 predispose to familial breast and ovarian cancer (FBOC). Additionally, biallelic mutations in FANCD1/BRCA2 and FANCN/PALB2 are associated with neuroblastoma, medulloblastoma, and Wilms Tumor [129]. FANCA and FANCC with higher mutation rate are associated with breast cancer, ovarian cancer, cervical cancer, pancreatic cancer, and other malignancies (Fig. 1) [127].

Although FA is associated with various solid tumors, head and neck squamous cell carcinomas (HNSCCs) are the most frequently diagnosed tumors in FA patients. The risk of HNSCCs in FA patients is 500- to 700-fold higher than that in normal subjects, and the risk of other SCC such as esophageal and vulvar SCC is thousands of times higher than that in normal subjects [125].

**Fig. 6:**
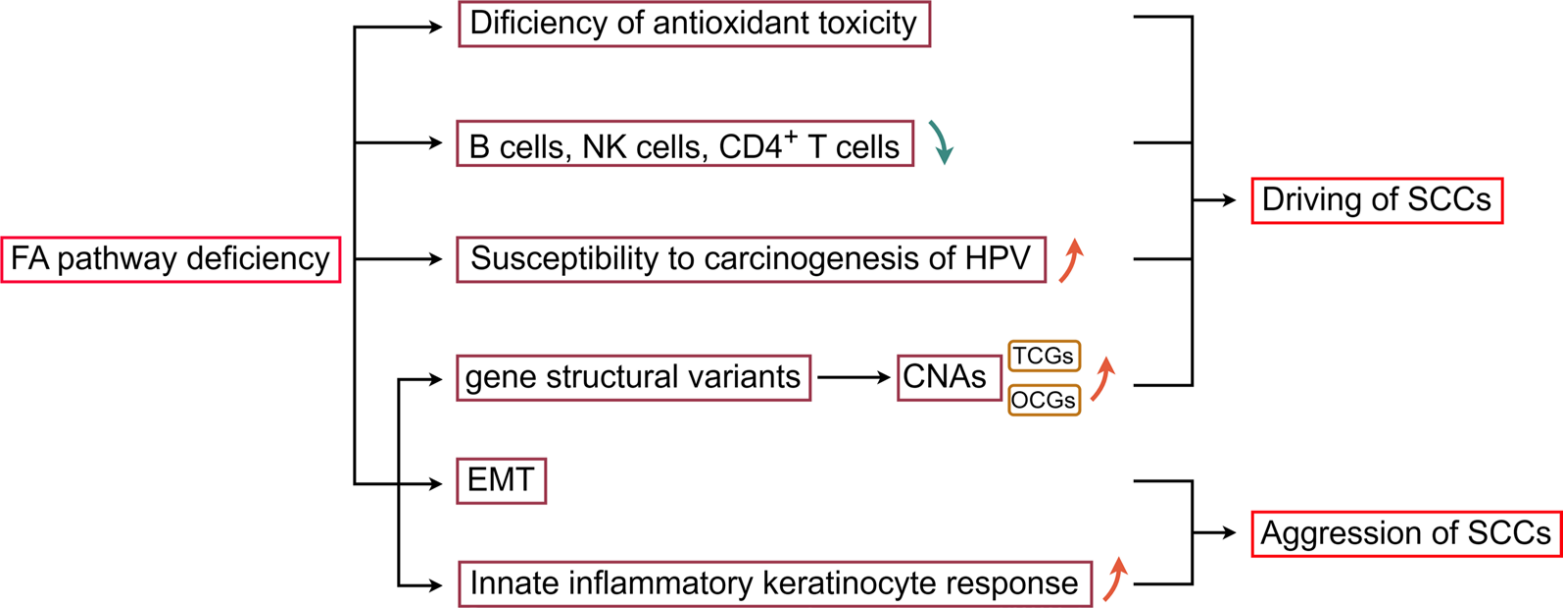
Mechanisms of FA pathway deficiency leading to SCCs: **①** FA genes have protective function against oxygen toxicity; **②** B cells, NK cells and CD4^+^ T cells are decreased in FA patients, which affects the body's immune surveillance of cancer cells; **③** Susceptibility to carcinogenesis of HPV are increased in FA patients; **④** Recent research advance in the molecular level of FA SCCs, the deficiency of FA repair pathway causes gene structural variants, somatic CNAs of oncogenes and tumor suppressor genes are increased, driving the development of SCCs, additionally EMT and increase of innate inflammatory keratinocyte response promote the aggressive of SCCs. *FA, Fanconi anemia; CNAs, copy number alterations; OCGs, oncogenes; TSGs, tumor suppressor genes; EMT, epithelial-mesenchymal transition; HPV, human papillomavirus; SCCs, squamous cell carcinomas*

Currently, specific FA gene mutations associated with HNSCCs have not been identified. The reasons for the significantly increased risk of SCC in FA patients and the related mechanisms are not yet clear (Fig. 6): (1) Some studies have shown that low oxygen concentration in the environment can eliminate chromosome breaks in FA cells. Combined with the fact that the preferred anatomical site of SCC in FA patients usually involves the area exposed to atmospheric oxygen, some scholars have proposed that FA-related genes have a protective function against oxygen toxicity [130]. However, there is no clear evidence supporting this hypothesis; (2) Some studies find B cells, NK cells and CD4+T cells in FA patients are all reduced. Therefore, scholars have hypothesized that the reduction of the cells in FA patients impairs immune function, which affects the body's immune surveillance of cancer cells [131]; (3) FA patients may have increased susceptibility to the carcinogenic effects of human papillomavirus (HPV) [132]; (4) Recent genomic and exome sequencing results of FA SCCs patients indicate that these patients have low rates of HPV infection, but high frequencies of TP53 mutations. Defect in the FA repair pathway lead to abundant structural variations in the genome, somatic copy number alterations (CNAs) in oncogenes (OCGs) and tumor suppressor genes (TSGs), driving the development of SCCs. Additionally, it also leads to epithelial-mesenchymal transition (EMT) and increased innate inflammatory keratinocyte response, enhancing the invasiveness of FA SCCs [20]. While there has been some progress in molecular-level research on FA SCCs, further studies are still needed to elucidate the pathogenic mechanisms and develop better treatment strategies for these patients.

### Epigenetic changes of FA genes and tumors

Epigenetic changes may result in abnormal expression or silencing of the genes, affecting DNA repair capacity and genomic stability in cells. Studies have shown that epigenetic alterations in FA genes can increase the risk of hematological malignancies or solid tumors. Some researchers propose that hypermethylation of promoter regions of FA gene can lead to the inactivation of FA protein, affecting the function of the FA pathway, thereby promoting tumor formation. For instance, high methylation of the FANCF (observed in a leukaemic CHRF-288 cell line), FANCC, and FANCL have been reported in a small portion of primary AML and acute lymphoblastic leukemia (ALL) cases [133]. Similarly, high FANCF methylation has been detected in solid tumors such as ovarian cancer, cervical cancer, oral cancer, and non-small-cell lung cancer. However, studies from Japan and China have reported that the methylation rate of the FANCF gene promoter is very low or even absent in breast cancer and gastric cancer patients [134, 135]. Recently, research has shown that FANCF in colorectal cancer tissue exhibits lower methylation levels as determined by the qMSP method with higher accuracy. It is suggested that FANCF hypomethylation might lead to FANCF overexpression, disrupting the FA pathway and consequently contributing to cancer development [136]. Whether the different results are related to the methylation detection methods remains to be clarified. Moreover, it is necessary to explore the specific molecular mechanisms by which epigenetic alterations, such as methylation of FA genes, drive tumor development and to identify potential therapeutic targets. Studying the epigenetic changes of FA genes will help reveal the mechanism of gene inactivation in the absence of mutations and expand our understanding of functional defects in the FA pathway.

## Conclusions

Various endogenous and exogenous crosslinking agents induce DNA ICLs, which are primarily repaired by the precise FA/BRCA pathway. Additionally, there are faster repair pathways, such as the NEIL3-mediated pathway for AP-ICLs, psoralen-ICLs, and the REV1-mediated pathway for acetaldehyde-ICLs, although some key enzymes in these pathways remain unclear. Mutations or epigenetic changes in FA-related genes affect FA protein activity, thereby impairing the function of the FA repair pathway. Failure in FA repair pathway results in bone marrow failure, congenital malformations, and cancer susceptibility. At present, bone marrow failure has been significantly improved by HSCT, gene therapy and other treatments. The challenge lies in the treatment of hematological or solid tumors. Because FA patients are highly sensitive to crosslinking agents, the toxicity of radiotherapy or chemotherapy is enhanced, leading to poor tolerance to anticancer treatments. Although recent molecular studies have shed light on FA-related tumors, especially highly prevalent HNSCCs, further investigation into the specific mechanisms underlying tumor development is still needed to identify more effective therapeutic targets or treatment strategies to improve patient survival.

## Data Availability

Not applicable.

## Abbreviations

ADH5: Alcohol dehydrogenase 5
ALDH2: Aldehyde dehydrogenase 2
ALL: Acute lymphoblastic leukemia
AML: Acute myelogenous leukaemia
AP: Apurinic/apyrimidinic
ATR: Ataxia-telangiectasia and Rad3-related protein
BER: Base excision repair
BIR: Break-induced replication
BLM: Bloom
BTB: Bric-a-brac, Tramtrack, Broad complex
BTR: BLM-TOPO3α-RMI1-RMI2
CHK1: Checkpoint kinase 1
CK2: Casein kinase 2
CMG: CDC45-MCM2-7-GINS
CNAs: Copy number alterations
CtIP: CtBP-interacting protein
dA: Deoxyadenine
dC: Deoxycytosine
dG: Deoxyguanine
DHJ: Double Holliday junction
DNA2: DNA synthesis defective 2
DSB: Double-strand break
EMT: Epithelial-mesenchymal transition
Et: Ethylidene
EXO1: Exonuclease 1
FA: Fanconi anemia
FBOC: Familial breast and ovarian cancer
HNSCCs: Head and neck squamous cell carcinomas
HPV: Human papillomavirus
HR: Homologous recombination
HSCT: Hematopoietic stem cell transplantation
ICL: Interstrand crosslink
Ile44: Isoleucine 44
MCM2–7: Mini-chromosome maintenance 2–7
MDS: Myelodysplastic syndrome
MLR: MUS312/MEI9 interaction-like region
MMC: Mitomycin C
MRN: MRE11-RAD50-NBS1
NEIL3: Endonuclease VIII-like 3
NER: Nucleotide excision repair
NGS: Next-generation sequencing
NHEJ: Non-homologous end joining
NO: Nitric oxide
OCGs: Oncogenes
OHMe: Hydroxymethyl
PARP: Poly-ADP-ribose polymerase
PCNA: Proliferating cell nuclear antigen
PdG: Propano-2'-deoxyguanosine
PLK1: Polo-like kinase 1
pre-RCs: Prereplication complexes
qMSP: Quantitative methylation-specific PCR
RECQ1: RecQ like helicase 1
RPA: Replication protein A
SCC: Squamous cell carcinoma
SMC: Structural maintenance of chromosomes
SRA: SET and RING finger associated
ssDNA: Single-stranded DNA
TLS: Translesion synthesis
TOP1: Topoisomerase I
TRAIP: Traf-interacting protein
TSGs: Tumor suppressor genes
UAF1: Ubiquitin associated factor 1
UBZ: Ubiquitin-binding zinc finger
UHRF1: Ubiquitin-like with PHD and RING finger domains 1
USP1: Ubiquitin specific peptidase 1
WRN: Werner syndrome protein
